# Polymer Structural Composites Reinforced with Hemp Fibres—Impact Tests of Composites After Long-Term Storage in Representative Aqueous Environments and Fire Tests in the Context of Their Disposal by Energy Recycling Methods

**DOI:** 10.3390/polym17030276

**Published:** 2025-01-22

**Authors:** Mieczyslaw Scheibe, Renata Dobrzynska, Magdalena Urbaniak, Andrzej Bledzki

**Affiliations:** 1 Faculty of Marine Engineering, Maritime University of Szczecin, 70-500 Szczecin, Poland; mscheibe@vp.pl; 2 Faculty of Maritime Technology and Transport, West Pomeranian University of Technology in Szczecin, 71-065 Szczecin, Poland; renata.dobrzynska@zut.edu.pl; 3 Faculty of Mechanical Engineering and Mechatronics, West Pomeranian University of Technology in Szczecin, 70-310 Szczecin, Poland

**Keywords:** natural plant fibres, polymer structural composites, industrial hemp (*Cannabis sativa* L.), hemp fibres, disposal, recycling

## Abstract

This paper presents the potential for an alternative use of structural polymer composite reinforcement, made from natural industrial hemp (*Cannabis sativa* L.) fibres, in the manufacture of selected products in the shipbuilding industry. This research used fabrics made from unmodified and chemically modified industrial hemp fibres. The primary research focus was on determining the impact strength of the new eco-friendly structural composites produced after long-term storage in representative aqueous environments. Also presented are the results of fire response tests of these composites in the context of their disposal by energy recycling. The tests carried out also referred to a well-defined glass fibre-reinforced polymer composite, from which a control slab of the actual product was realistically produced in the form of a representative section of a 34-foot boat hull plate below the waterline. The results of this basic research into these structural composites confirmed the validity of continuing, respectively, application and implementation research, aimed at producing composites dedicated to selected products of the shipbuilding industry.

## 1. Introduction

Over the past 30 years, there has been significant interest in various industries, for economic and environmental reasons, in the potential use of natural plant fibres (NPFs) in polymer composite materials because of their environmentally friendly impact and global economic sustainability. A significant increase in environmental risk has been observed on our planet, resulting, inter alia, from a faster-than-expected massive increase in waste post-production waste of non-biodegradable plastics used in the chemical and automotive (mainly automotive) industries, as well as hard-to-recycle, end-of-life (EOL), large-scale engineering products (wind turbine blades, recreational vessels and many others), manufactured in dedicated specialised applications from polymer composites [1,2].

The increasing environmental threat has led to research on using NPF as an alternative to synthetic glass fibres (GFs) in polymer composites. Due to the availability of common NPFs in almost all geographical regions of the world and the relative ease with which plant fibres can be extracted from them (sisal, ramie, flax, hemp), the attention of scientists has been particularly focused on these plants. Since the late 1990s, the related specialist literature from around the world has begun to record the results of research work carried out in the area of methods for processing fibres and their modification, with research into the mechanical properties and industrial use of NPFs, and in 2001–2004 in particular, hemp fibres (HFs) as an alternative to GF reinforcement in polymer composites were studied [3,4,5]. From 2006 to 2011, the optimisation of the properties of HFs used as a reinforcement in polymer composites and the properties of these composites were investigated at research and development centres on different continents (North America, Europe, South-East Asia). The properties of polymer composites with HFs were investigated, optimising fibre properties using novel defibring and fibre characterisation methods. A study was conducted on the mechanical treatment of HF-based bast fibres in the use of applied fibre extraction and separation processes for textile-reinforced composites, which caused significant damage to these fibres. The effect of fibre defects on their mechanical properties and their susceptibility to chemical degradation was determined. Systematic and improved methods are presented for the characterisation of surface and fracture of elemental HF using field emission scanning microscopy (FE-SEM), the determination of microfibre angles (MFAs) using advanced microscopy technology, and the study of crystallinity by X-ray diffraction (XRD) and Fourier transform infrared (FTIR) [6,7]. A study on the impact and fatigue behaviour of HF composites was carried out to characterise the post-impact tensile and fatigue properties of a non-woven polyester mat reinforced with HF. In addition, the degradation of the tensile modulus of elasticity during fatigue cycling was investigated [8,9,10]. In 2012, research continued in Europe in the area of the effect of treating natural HFs on the long-term thermal properties of PP/HF (polypropylene/hemp fibre) composites, as well as in the area of predicting the moduli and strength of composites reinforced with these fibres [11,12,13]. Also, in the same year, the flexural properties of HF-reinforced polylactide and unsaturated polyester composites and various chemically treated unsaturated HF-reinforced polyester composites were investigated at research and development centres in Europe, Australia and New Zealand, in the context of the mechanical quantities (Young’s modulus, strength and impact strength), and the dependence of Young’s modulus (E) on HF diameter was investigated by determining interdependent ultrastructural parameters such as cellulose crystallinity and microfibre angles. In addition, factors and conditions affecting the augmentation of the mechanical properties of the components were presented on the basis of subsequent studies [14,15,16,17]. From 2013 to 2021, research in Europe continued to show that HF-reinforced composites with thermoplastic matrices, thermosetting matrices and biodegradable matrices have good mechanical properties, and when used to improve the fibre/matrix interfacial bond, chemical treatments of the HF surface resulted in significant improvements in the mechanical properties of the composites [18,19]. The impact of polypropylene biocomposites reinforced with short HF was investigated, using an impact machine equipped with a camera for the rapid imaging and measurement of macro-crack propagation for mechanical measurements. The impact behaviour was analysed using the finite element method (FEM) [20]. The effects of fibre length and alkali and silane treatments on the mechanical and physical properties of HF-reinforced polypropylene composites were also investigated. A compression moulding technique was used to produce the composite. Longer fibres were shown to increase the mechanical strength of the PP/HF composite [21,22,23]. Composites reinforced with NPF, with a particular emphasis on industrial hemp (*Cannabis sativa* L.) fibres, is currently a rapidly growing mechanical engineering discipline in the science of structural polymer composites. With respect to the GF used, HF are low-cost, low-density fibres, are biodegradable and exhibit satisfactory specific properties and physical and mechanical parameters. Structural polymer composites based on them are in many respects comparable to common GFRP.

Interest in this issue has also emerged in Poland, particularly in the use of structural polymer composites in shipbuilding and peri-shipyard production [24,25,26,27].

Based on the generally available test results of HFs in relation to type ‘E’ GFs, a comparison of the general properties of these fibres is presented in Table 1.

It should be noted that the conclusions in Table 1, resulting from the comparison of the physical–mechanical properties, environmental properties and economic condition of the tested fibres, clearly indicate the many advantages of HFs, which include an approximately 2× lower density (lower weight of the future technical product), the high elasticity of the surface and internal structure of the composite, an ability to absorb impact, and resistance to open cracking. The economic aspect is also important: easy availability and low price of the raw material and low energy required to produce 1 tonne of fibre. What is essential and disproportionately important is the environmental aspect in terms of the possibility of disposal by energy recycling.

Cellulose fibre-based composite materials have remarkable economic and environmental advantages, such as low cost, easy market availability, low density, high strength-to-weight ratio, acceptable strength properties and biodegradability (environmental friendliness).

In order to test and confirm both the advantages and disadvantages of NPF-reinforced composite materials, the first stage of this research focused on carrying out the chemical modification of industrial hemp fibres contained in hemp fabric. Based on an analysis of available scientific publications thematically related to the issue of the chemical modification of HF, it was assumed that the most favourable process would be to refine hemp fibres by alkalisation, using sodium hydroxide (NaOH—r-r-r 22%). The alkalisation of the fibres of the 100% hemp fabric formats consisted of immersing these formats in a tank of solution and applying NaOH to the fabric fibres over a period of 30 min at room temperature (21 ± 0.5 °C). After this time, the alkalisation process was interrupted and the formers were subjected to rinsing at intervals of 10 min in three successive tubs of demineralised water at 21 °C until complete removal of NaOH and reaching pH ~7. The fabric rinsing process was then stopped and the formers were subjected to natural drying (humidity 54 ± 2%, air temperature 19 ± 1 °C) for 72 h. The alkalinisation process removed the cuticle, which is the epidermal (epidermis) layer of the cellulose fibre, facilitating the diffusion and re-reactivity of the cellulose molecules with the chemical modifier. The final effect of NaOH was the modification of HF elemental cells, which, through the chemical process of NaOH permeation into the crystalline areas of the fibre cellulose, resulted in the formation of alkalicellulose and, after washing the unreacted NaOH three times in demineralised water, led to the production of regenerated cellulose. It should be noted that there is a fundamental difference in properties between regenerated and natural cellulose, as there is a far-reaching degradation of the fibre structure during the dissolution process, with the regenerated product tending to be less crystalline. Regenerated cellulose is not soluble in water, and has good mechanical properties and good barrier properties against gases (except water vapour). In order to reduce water vapour permeability, products made from regenerated cellulose are coated with a barrier layer, e.g., vinylester resin, and when used in polymer structural composites, a standard DCPD construction resin (so-called enhanced polyester resin). The second stage of the research focused on the production of new composites using the hand lay-up (HLU) method. The composites, **HFRP** (hemp fibre-reinforced polymer) and **HFRPm** (hemp fibre-reinforced polymer with chemically modified hemp fibres), respectively, were prepared using unsaturated polyester DCPD (DiCykloPentaDien) as the matrix and HF and HFm in fabric form as their reinforcement and a GFRP-based composite reinforced with GF.

In the third stage of the study of the new composites, the physical (density and water absorption), mechanical (tensile, flexural, impact) and environmental (fire response tests) properties were determined in the context of the effect of chemical treatment on HF. In order to understand the fibre–matrix interaction in the newly produced composites, the morphology of the fractured surfaces of the composites was investigated using scanning electron microscopy (SEM). The results obtained are presented graphically and, based on the analysis of the results, related issues are clarified [24,25,27]. As HFs have a high environmental resistance, the produced **HFRP** and **HFRPm** composites, with respect to the GFRP composite, can be legitimately and purposefully used as biodegradable composites with favourable physical properties in the production of selected structures. In addition, due to their uncomplicated manufacturing process, significantly shorter processing times and lower production costs, these composites can be used as a versatile material in the field of mechanical engineering and manufacturing technology for selected structural products [26,27].

Due to the lack of published results on the impact testing (Charpy method) of manufactured hemp fibre-reinforced polymer composites, a study was undertaken to determine the effect of the content of NaCl [‰] and other trace elements in the respective waters on the transfer of violent dynamic loads by the tested materials, after three months of their storage in aqueous environments with varying degrees of water salinity. In addition, tests were carried out on the reaction-to-fire performance of the **HFRP** and **HFRPm** composites vs. the GFRP composite in the context of quasi-complete disposal by energy recycling.

## 2. Materials and Methods for Producing Composites for Testing

To test the impact performance of the new pro-environmental structural composites, **HFRP** (series K1–K5) and **HFRPm** (series Km1–Km5) polymer structural composites were produced after long-term storage in representative aqueous environments. A total of 10 control slabs were produced, reinforced with a variable number of layers of fabric made from 100% HF, a new eco-friendly material envisaged for alternative use in the construction of selected vessels.

Conducting a fire response test of **HFRP** and **HFRPm** material vs. GFRP, in the context of their disposal by energy recycling, required the fabrication of an additional GFRP control slab (SE) of a real product in the form of a representative 34ft boat hull plate below the waterline, incorporating GF reinforcement.

### 2.1. Materials Used in the Study Composite Matrix Material

#### 2.1.1. Matrix Material (Appendix A.1)

1. DCPD standard construction resin (so-called improved polyester “yacht” resin);
2. Initiator for the copolymerisation;
3. Pro-adhesive agent;
4. Polymerisation accelerator based on cobalt octanoate 6% (dissolved in xylene).

#### 2.1.2. Composite Reinforcement Material (Appendix A.2)

1. A 100% hemp fabric with a 1/1 “plain” weave, produced by weaving from threads made from long-fibre hemp (*Cannabis sativa* L.);
2. GF sewn mats.

### 2.2. Method of Producing Composites for Testing

Composites in the form of control slabs were made by hand lamination (HLU) by experienced and specialised production staff.

Prior to the fabrication of the control slabs, all GFs as well as the HF and HFm slabs were seasoned under production conditions in a thermal chamber with convection heat circulation at 60 °C for 24 h. Appropriately sorted and labelled format packs were prepared for the manufacture of control slabs (Table 2).

The method of chemical modification of the reinforcement formats (HFms), as well as the production of GFRP, **HFRP** and **HFRPm** composites and the preparation of test samples from them is described in detail in [24,27].

Control slabs of structural polymer composites GFRP, **HFRP** and **HFRPm** were made in the production hall of TTS (Technologie Tworzyw Sztucznych Sp. z o.o. in Łozienica near Goleniow, Poland—manufacturer of sailing and motor yachts and motor boats), under the supervision of a quality control officer maintaining the conditions according to points 4.2.2–4.2.4 of the Polish Register of Shipping No. 1/1998 [33]. The number of reinforcement layers was based on the assumptions in [24].

In order to determine the format grammage of the reinforcement package (number of reinforcement layers) of the composite material of the specified control slabs, all GFs and the HF and HFm formats were dimensioned, weighed and permanently numbered accordingly: format GF (SE); format HF (K1–K5); HFm (Km1–Km5) (Table 3).

In addition, in order to test the fire response of the **HFRP** material and **HFRPm** vs. GFRP, in the context of their disposal by energy recycling, the thicknesses of the samples produced from the control slabs were measured (Table 4).

## 3. Research Methods and Results

In the literature review of available and published scientific papers, research publications, research reports, etc., worldwide/in Europe/in Poland, no descriptions of previously conducted or completed impact tests of polymer structural composites in various aquatic environments, including composites reinforced with natural plant fibres, in particular hemp fibres, were found. Hence, this initiated the idea of conducting such tests in a natural and real environment for newly produced construction materials reinforced with these fibres for use in the construction of floating units, i.e., in the environment of use of these units manufactured in the shipbuilding sector of the shipbuilding industry. Additionally, the reaction to fire of the **HFRP** and **HFRPm** materials vs. GFRP material of the hull of a 34-foot boat was investigated in the context of the disposal of these materials using the energy recycling method.

### 3.1. Impact Strength Testing (Charpy Method) of Composites in Representative Aqueous Environments

The aim of the study was to determine the ability of new **HFRP** and **HFRPm** polymer structural composites to withstand rapid dynamic loads in their natural operating conditions (in waters with different degrees of salinity). The unprotected edges of the samples after cutting out from the control slabs were covered with a micron protective layer of DCPD matrix material. Samples of the tested materials were stored in representative centres: (1)—demineralized water; (2)—fresh water (samples: Lake Miedwie; Poland, Żelewo—geographical coordinates: 53.288862, 14.869285; August 2021); (3)—brackish water—with salinity of 7.8‰ (samples: Baltic Sea; Poland, Dziwnów—geographical coordinates: 54.029464, 14.762658; August 2021); (4)—salty water—with salinity of 38‰ (samples: Adriatic Sea; Croatia, Verudela Beach—geographical coordinates: 44.833609, 13.832109; August 2021) for 3 months [34]. Samples of the new **HFRP** (K1–K5) and **HFRPm** (Km1–Km5) material and the GFRP (SE) hull structure material of a 34-foot boat were subjected to Charpy impact testing in accordance with ISO 15314 (Methods of exposure to sea: Method C involving exposure in which the samples are completely immersed in water—five pieces each with dimensions of 80 × 10 × thickness mm) [35]. The tests were carried out on standard unnotched samples using a pendulum hammer manufactured by VEB Werkstoffpruefmaschinen Leipzig-Betrieb des VEB “Fritz Heckert”/German Democratic Republic with a maximum impact energy of up to 50 J and up to 300 J. Conducting a three-month storage test of composites was possible while maintaining appropriate water conditions and quality conditions in these aquatic environments.

The test was conducted in a closed room with a constant temperature of 22 °C and air humidity of 60 ± 1%, in open containers filled with these waters. The samples were completely immersed in separate containers with waters, in which the levels of these waters were replenished every 7 days to their initial state. Each time the level of these waters decreased after this time was the result of surface evaporation of water in the containers. At the end of each passing month, the containers were emptied of water and refilled with “new/fresh” water. This procedure was intended to eliminate water bloom, i.e., a volumetric change in colour in the aquatic environment caused by the mass development of microscopic living organisms that are unrecognisable to the naked eye.

### 3.2. Analysis of the Results of Impact Tests (Charpy Method) of Materials in Representative Water Environments

The analysis of the impact strength test results (Charpy method) was aimed at determining the effect of the NaCl [‰] content and other trace elements in individual waters on the transfer of sudden dynamic loads by the tested materials, which could be used in potentially exploited composite floating objects.

**Figure 1:**
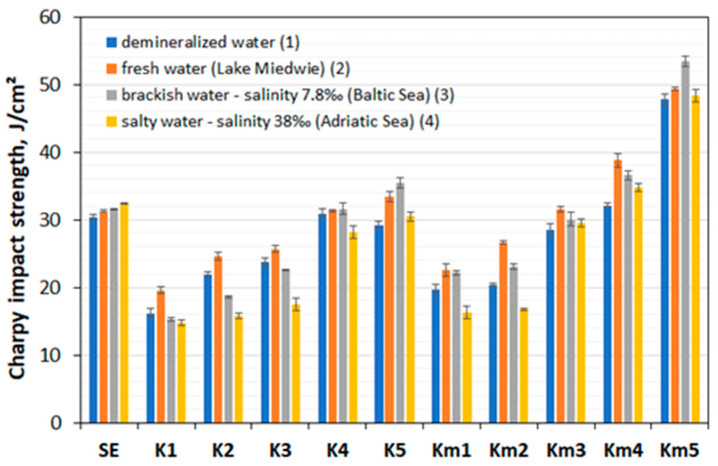
Charpy impact strength comparison table of **HFRP**, **HFRPm** and GFRP composites after 3 months of their storage in representative aqueous environments (**1**)–(**4**).

The results of the impact strength tests (Charpy method) of composite samples of individual series K1–K5 and Km1–Km5 vs. series SE material after 3 months of their storage in aqueous environments (**1**)–(**4**) are presented in Table 5 and Figure 1.

In order to illustrate the analysis of the test results, a comparative graph of the impact strength (Charpy method) of the tested materials is provided (Figure 1).

The storage of the polymer composite samples **HFRP** and **HFRPm** in layers, in natural aqueous environments, was as follows: K4–K5 series (9–11) and Km3–Km5 series (7–11) in the environment (**2**), K4–K5 series (9–13) and Km4–Km5 series (9–11) in the environment (**3**) and Km4–Km5 series (9–11) in the environment (**4**); these layers show the higher impact strength values of the composite samples in comparison to the SE series samples of the GFRP composite.

The test results for the SE series GFRP show a consistent slight increase in impact strength (Charpy method) in aqueous environments (**1**)–(**4**). Similar test results in aqueous environments (**2**)–(**3**) were also obtained by the K1–K5 series of composites. Considering the test results referring to the conditions of individual water environments (**2**)–(**4**), i.e., natural environments in which floating objects are operated, a constant increase in impact strength is noticeable: in the water environment (**2**), the K4 and K5 series at the level of 11% and the Km4–Km5 series at the level of 33% vs. the SE series; similarly, in the water environment (**3**), the K4 and K5 series at the level of 12% and the Km4–Km5 series at the level of 42%. In the aqueous environment (**4**), in relation to the SE series, the K4 and K5 series show a decrease in impact strength of 13%, and the Km4–Km5 series show an increase in impact strength of 28%.

It should be noted that the **HRFP** composites of the K4–K5 series show a steady slight increase in the impact strength of these new materials in the (**2**) and (**3**) environments, while the **HFRPm** composites of the Km3–Km4 series in the (**3**) environment show an average decrease in impact strength of 6%, and in the (**4**) environment, a decrease of 7% with respect to the impact strength of these materials in the (**2**) environment. At this stage of the analysis, the research results confirm the possibilities of using the new **HFRP** composites of the K4 and K5 series and **HFRPm** of the Km4 and Km5 series, e.g., in the HLU production of selected products in the shipbuilding sector, meeting the limited requirements for medium- and low-strength floating object components.

### 3.3. Fire Reaction Tests of New HFRP/HFRPm Polymer Composites

The preliminary tests were carried out in accordance with the research methodology specified in ISO 5660 [36]. The standard specifies a measurement method for the heat release rate and smoke production rate of specimens exposed in the horizontal orientation to irradiance. The heat release rate is determined by measurement of the oxygen consumption derived from the oxygen concentration and the flow rate in the combustion product stream. The time to ignition (sustained flaming) is also measured in this test. The dynamic smoke production rate is calculated from the measurement of the attenuation of a laser light beam by the combustion product stream. Smoke obscuration is recorded for the entire test, regardless of whether the specimen is flaming or not. Horizontally placed samples of 100 mm in width and 100 mm in length and thickness, respectively, for GFRP laminate (7.50 mm), **HFRP** (13.85 mm) and **HFRPm** (21.95 mm), were exposed to a 50 kW/m^2^ irradiance. The ignition of samples was triggered by a spark igniter.

### 3.4. Analysis of the Results of Fire Reaction Tests of New HFRP/HFRPm Polymer Composites

**Figure 2:**
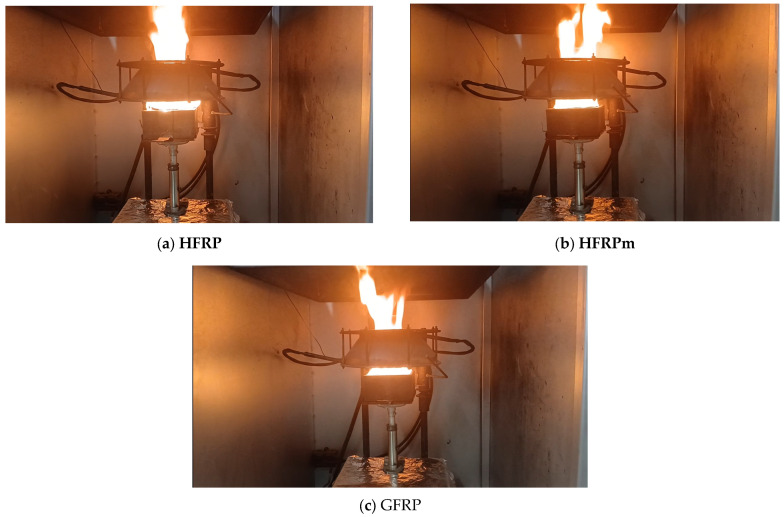
Photos of samples in the course of ignition: (**a**) **HFRP**; (**b**) **HFRPm**; (**c**) GFRP.

Photos of samples **HFRP**, **HFRPm** and GFRP composites in the course of ignition are shown in Figure 2. The obtained results of preliminary tests of the materials are compared in Table 6.

**Figure 3:**
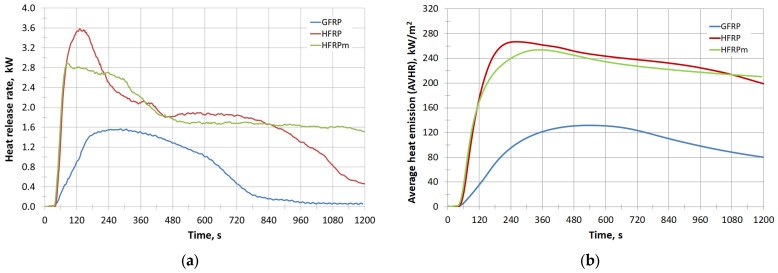
(**a**) Heat release rate with time; (**b**) average heat emission (AVHR) with time.

**HFRP** and **HFRPm** samples burn longer and more efficiently than GFRP—their combustion is almost complete. **HFRP** samples burned by approx. 96%, and **HFRPm** by approx. 83%. This is a significant difference compared to GFRP, whose residues after combustion are approx. 53%, which may cause a greater problem with waste management after subjecting this material to neutralisation through heat recovery. During this process, more heat can be obtained from **HFRP** and **HFRP** materials than from GFRP, as indicated by the results of measurements of the heat release rate (Figure 3a), average rate of heat emission (Figure 3b) and total heat release during combustion of the tested materials (Table 6). The most heat during combustion is released by **HFRPm** (252 MJ/m^2^), with **HFRP** at about 239 MJ/m^2^, and GFRP at only 96.2 MJ/m^2^.

**Figure 4:**
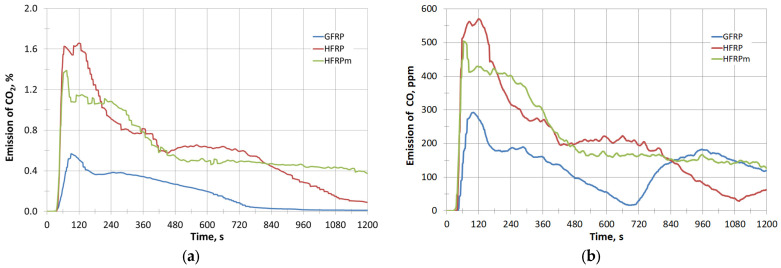
(**a**) Emission of CO_2_ with time; (**b**) Emission of CO with time.

Based on the obtained test results, it can also be stated that **HFRP** and **HFRPm** samples emit larger amounts of carbon monoxide and carbon dioxide during combustion compared to GFRP (Figure 4a,b). However, when calculated per unit of material mass, the CO emission values for **HFRPm** and **HFRP** are lower than that of GFRP. The specific CO emission values are 0.116, 0.144 and 0.153 kg/kg, respectively.

## 4. Conclusions

Impact test results (Charpy method) of new polymer composites of the series K1–K5 and Km1–Km5 vs. series SE, after 3 months of storage in representative aqueous environments (**1**)–(**4**), are presented in Table 5.

The results clearly show that the previously commonly used GFRP composite slightly increases its impact strength with growing salinity of the natural aqueous environment (**2**)–(**4**). In addition, the series K4–K5 and Km4–Km5 composites, under the same conditions of the aqueous environment with the exception of salt water with a salinity of 38‰ (**4**), show an increase in impact strength up to 6 and 8%, respectively. Also, it has been noted that the series K4–K5 and Km4–Km5 show an increased impact strength in fresh water (**2**) and in saline water with salinity of 7.8‰ (**3**) with respect to the GFRP composite.

The results of the fire response testing of the new polymer composites **HFRP** and **HFRPm** compared to GFRP, in light of the need to meet the current sustainability requirements of worldwide economies and to meet increasingly stringent environmental requirements, show that these composites, respectively, can and should be quasi-fully recycled through the method of energy recycling.

The **HFRPm** composite gives off the most heat during combustion (2.62), while **HFRP** gives off 2.48 times more heat than GFRP. In addition, it was noted that the waste from controlled combustion (by weight of the composite materials tested) decreased significantly and remained as follows: **HFRP**—3.7; **HFRPm**—17.4; GFRP—52.7%. It is therefore advisable to use the waste **HFRP/HFRPm** materials as an energy fuel and the residue as ash as a full component of effective natural fertilisers. On the basis of the test results obtained, it was also observed that the **HFRP** and **HFRPm** emit higher amounts of carbon monoxide (CO) and carbon dioxide (CO_2_) during combustion compared to GFRP. However, per unit mass of material, the CO emissions for **HFRPm** and **HFRP** are lower than that of GFRP (Table 6).

The results of physical, environmental and mechanical properties, with particular emphasis on the impact strength (Charpy method) of the series K1–K5 and Km1–Km5, after 3 months of storage in representative aqueous environments (**1**)–(**4**), are presented together with their assessment of their applicability to shipbuilding in Table 7.

The experimental results of physical and mechanical tests of the selected **HFRP** composites of the K4 and K5 series and the **HFRPm** composites of the Km4 and Km5 series in relation to the GFRP composite of the SE series, collected and presented in a comparative table (Table 7), indicate the validity of the alternative use of HF/HFm reinforcement instead of the commonly used E-type GF reinforcement.

The homogeneity of the geometric shape and the reproducible structure of the GF, ensured by the chemical manufacturing process, results in reproducible physical–mechanical and morphological properties of these fibres. Different in structure, with respect to GF, are the non-uniform geometric shape and uneven wall thickness of NPF of HF, which are characterised by a non-uniform structure (porosity) of the plant material. Despite the use of the same type of matrix (standard DCPD engineering resin) in the structural polymer composites, the demonstrated difference in their strength parameters is derived from the difference in the structure of the NPF in relation to the GF structure.

The reduction in some of the indicated performance values of the tested **HFRP** and **HFRPm** composites compared to the GFRP composite allows them to be used in the shipbuilding industry, to a limited extent, in the HLU process for the manufacture of shell components with medium and low strength requirements. The results obtained during this basic research on these structural composites clearly confirmed the need for application research and the desirability of carrying out implementation research to obtain composites dedicated specifically to selected products manufactured in the shipbuilding industry.

The use of new **HFRP** composite structures (reinforced alternatively with HF only, or with varying amounts of HF and GF or CF (carbon fibres)—so-called hybrid reinforcements)—in the shipbuilding industry for the hull plating of selected vessels appears to be an appropriate alternative to the composite watercraft currently manufactured from GFRP and fits in with the programme for implementing the recommended and EU-required environmentally friendly measures in the production economy of the global shipbuilding industry.

## Figures and Tables

**Table 1:** Comparative table of the general properties of HFs and GFs (own elaboration [27] in combination with [28,29,30,31,32]).

Properties	HF	GF
Density, g/cm^3^	1.4–1.5	2.5
Young’s modulus, GPa	70	70
Tensile strength, MPa	550–900	2000–3500
Elongation at break, %	1.6	2.5–3.0
Flexibility	big	small
Ability to absorb impact	big	small
Resistance to open cracking	big	small
Surface cracks	flexible, soft	brittle, sharp
Corrosion resistance	yes	yes
Moisture absorption, %	8	-
Necessity of chemical modification	no/yes	no
Resistance to very high temperatures	mean	high
Renewability	yes	no
Harmful to health	small	big
Environmental benefits:		
-biodegradability	yes	no
-energy recycling	yes	no
Economic benefit: price of 1 tonne of raw material/mat/fabric, USD	1000–1900	1600–3300
Energy required to produce 1 tonne of fibre, GJ	4	30

**Table 2:** Designation of control slabs/sample series depending on the number of reinforcement layers.

Number of Reinforcement Layers	Designation of a Series of Samples
GF—E-glass—8 layers	SE
HF—unmodified hemp—3 layers	K1
HF—unmodified hemp—5 layers	K2
HF—unmodified hemp—7 layers	K3
HF—unmodified hemp—9 layers	K4
HF—unmodified hemp—11 layers	K5
HFm—modified hemp—3 layers	Km1
HFm—modified hemp—5 layers	Km2
HFm—modified hemp—7 layers	Km3
HFm—modified hemp—9 layers	Km4
HFm—modified hemp—11 layers	Km5

**Table 3:** Grammage of the reinforcement package used to make the control slabs.

Designation of the Sample Series/Number of Reinforcement Layers	Grammage the Package of Formats, g/m^2^										
	SE	K1	K2	K3	K4	K5	Km1	Km2	Km3	Km4	Km5
HF/HFm—3 layers	–	1434	–	–	–	–	2047	–	–	–	–
HF/HFm—5 layers	–	–	2404	–	–	–	–	3369	–	–	–
HF/HFm—7 layers	–	–	–	3352	–	–	–	–	4993	–	–
GF—8 layers	6148	–	–	–	–	–	–	–	–	–	–
HF/HFm—9 layers	–	–	–	–	4379	–	–	–	–	6138	–
HF/HFm—11 layers	–	–	–	–	–	5280	–	–	–	–	7252

**Table 4:** Thickness of control slabs.

Designation of the Sample Series/Number of Reinforcement Layers	Thickness of Control Slab, mm										
	SE	K1	K2	K3	K4	K5	Km1	Km2	Km3	Km4	Km5
HF/HFm—3 layers	–	3.91	–	–	–	–	4.75	–	–	–	–
HF/HFm—5 layers	–	–	4.67	–	–	–	–	7.45	–	–	–
HF/HFm—7 layers	–	–	–	6.16	–	–	–	–	11.02	–	–
GF—8 layers	7.50	–	–	–	–	–	–	–	–	–	–
HF/HFm—9 layers	–	–	–	–	9.17	–	–	–	–	17.15	–
HF/HFm—11 layers	–	–	–	–	–	11.45	–	–	–	–	19.51

**Table 5:** Average values of impact strength (Charpy method) of **HFRP**, **HFRPm** and GFRP composites after 3 months of their storage in representative aqueous environments (**1**)–(**4**).

Designation of a Series of Samples/Aqueous Medium	Charpy Impact Strength, J/cm^2^			
	Demineralized Water (1)	Fresh Water (Lake Miedwie) (2)	brackish Water—Salinity 7.8‰ (Baltic Sea) (3)	Salty Water—Salinity 38‰ (Adriatic Sea) (4)
SE	30.3	31.2	31.6	32.4
K1	16.1	19.5	15.2	14.7
K2	22.0	24.6	18.6	15.7
K3	23.7	25.7	22.6	17.5
K4	30.9	31.3	31.6	28.1
K5	29.2	33.4	35.4	30.5
Km1	19.7	22.6	22.2	16.3
Km2	20.4	26.7	23.0	16.7
Km3	28.5	31.5	30.2	29.5
Km4	32.1	38.8	36.6	34.7
Km5	47.8	49.3	53.4	48.4

**Table 6:** Results of fire reaction tests.

Parameter	Material Tested		
	GFRP	HFRP	HFRPm
Mass of the specimen, g	96.43	136.67	180.99
Specimen thickness, mm	7.50	13.85	21.95
Ignition time, s	26	34	30
Extinction time, s	914	-	-
Duration of the test, s	1200	1200	1200
Mass loss, g	45.63	131.56	149.47
Mass loss rate, g/s	0.95	1.29	1.91
Average mass loss rate, g/m^2^s	19.664	42.088	35.623
Maximum heat release rate HRR, kW/m^2^	177	405	328
Maximum average rate of heat emission MARHE, kW/m^2^	131.73	267.24	253.57
Total heat release, MJ/m^2^	96.2	239	252
Oxygen consumption, %	0.67	1.82	1.39
Light attenuation, %	52.68	60.34	57.65
Smoke production rate *SPR*, m^2^/s	0.086	0.102	0.098
Total smoke production, *SC*, m^2^	34.3	58.6	67.0
Maximum CO_2_ concentration, %	0.567	1.658	1.388
Maximum CO concentration, ppm	293	570	504
Carbon monoxide yield, kg/kg	0.153	0.144	0.116
Carbon dioxide yield, kg/kg	3.148	6.714	5.118
Waste—incineration residues, g	50.80	5.11	31.52
Waste—incineration residues, %	52.7	3.7	17.4

**Table 7:** Analysis of the physical and mechanical properties of the tested composites and the possibility of using environmentally friendly **HFRP** and **HFRPm** composites in the shipbuilding industry.

Test Type	Construction Polymer Composite	GFRP	HFRP	HFRPm	Possibility of Using Environmentally Friendly HFRP and HFRPm Composites in the Shipbuilding Industry
Physical	Density, g/cm^3^	1.6	1.2–1.3	1.1–1.2	Yes
	Water absorption, %	0.1	3.0–4.3	3.3–7.4	Conditionally yes
Environmental	Waste—incineration residues, %	52.7	3.7	17.4	Definitely yes
Mechanical	Tensile elongation, %	–	3.8–4.4	11.8–12.5	Yes
	Elongation under static bending, %	–	2.2–7.4	3.7–11.5	Definitely yes
	Maximum tensile forces, N	–	2439–9209	1538–5758	Yes
	Maximum static bending forces, N	–	135–1789	173–3300	Definitely yes
Charpy impact strength of composite after 3 months storage in representative aqueous environments	Charpy impact strength in aqueous environments (fresh water—Lake Miedwie), J/cm^2^	31.2	19.5–33.4	22.6–49.3	Definitely yes
	Charpy impact strength in aqueous environments (brackish water—salinity 7.8‰—Baltic Sea), J/cm^2^	31.6	15.2–35.4	22.2–53.4	Definitely yes
	Charpy impact strength in aqueous environments (saline water—salinity 38‰—Adriatic Sea), J/cm^2^	32.4	14.7–30.5	16.3–48.4	Yes

## Data Availability

The data presented in this study are available on request from the corresponding authors.

## Appendix A

### Appendix A.1. Matrix Material

1. DCPD with the trade name AropolTM M 604 TBR (Safety Data Sheet No. 000000154974—Ashland) manufactured by INEOS Composites/Porvoo/Finland;
2. Metox-50WR resin manufactured by Oxytop Sp. z o.o./Antoninek near Steszew, Poland;
3. Maleic anhydride (MAH), at a ratio of 3g per 100 g of resin;
4. The name BÜFA^®^-Accelerator Co 6 (prod. No. 742-0600) from BÜFA Composite Systems GmbH & Co. KG, Rastede, Germany.

### Appendix A.2. Composite Reinforcement Material

1. Its average weight according to the manufacturer, i.e., the company S.C. CAVVAS LIMITED S.R.L., Cluj-Napoca, Romania, was HF = 478 g/m^2^. At the production stage, the fabric was treated with an impregnating agent named TUBIGUARD 27 (manufactured and selected by BEZEMA AG, Montlingen, Switzerland;
2. Manufactured by KROSGLASS S.A., Krosno, Poland, used to produce a representative hull of a selected vessel.
